# Epidermal growth factor receptor gene copy number in 101 advanced colorectal cancer patients treated with chemotherapy plus cetuximab

**DOI:** 10.1186/1479-5876-8-36

**Published:** 2010-04-16

**Authors:** Carla Campanella, Marcella Mottolese, Anna Cianciulli, Angela Torsello, Roberta Merola, Isabella Sperduti, Elisa Melucci, Salvatore Conti, Maria Grazia Diodoro, Massimo Zeuli, Giancarlo Paoletti, Francesco Cognetti, Carlo Garufi

**Affiliations:** 1 Department of Medical Oncology, Regina Elena Institute, via E Chianesi 53, 00144 Rome, Italy; 2 Department of Pathology, Regina Elena Institute, via E Chianesi 53, 00144 Rome, Italy; 3 Department of Clinical Pathology, Regina Elena Institute, via E Chianesi 53, 00144 Rome, Italy; 4 Department of Statistics, Regina Elena Institute via E Chianesi 53, 00144, Rome, Italy; 5 Department of Medical Oncology, Regina Elena Institute, via E Chianesi 53, 00144 Rome, Italy

## Abstract

**Background:**

Responsiveness to Cetuximab alone can be mediated by an increase of Epidermal Growth factor Receptor (EGFR) Gene Copy Number (GCN). Aim of this study was to assess the role of EGFR-GCN in advanced colorectal cancer (CRC) patients receiving chemotherapy plus Cetuximab.

**Methods:**

One hundred and one advanced CRC patients (43 untreated- and 58 pre-treated) were retrospectively studied by fluorescence in situ hybridization (FISH) to assess EGFR-GCN and by immunohistochemistry (IHC) to determine EGFR expression. Sixty-one out of 101 patients were evaluated also for k-ras status by direct sequencing. Clinical end-points were response rate (RR), progression-free survival (PFS) and overall survival (OS).

**Results:**

Increased EGFR-GCN was found in 60/101 (59%) tumor samples. There was no correlation between intensity of EGFR-IHC and EGFR-GCN (p = 0.43). Patients receiving chemotherapy plus Cetuximab as first line treatment had a RR of 70% (30/43) while it was 18% (10/56) in the group with previous lines of therapy (p < 0.0001). RR was observed in 29/60 (48%) of patients with increased EGFR-GCN and in 6/28 (21%) in those without (p = 0.02). At multivariate analyses, number of chemotherapy lines and increased EGFR-GCN were predictive of response; EGFR-IHC score, increased EGFR-GCN and number of chemotherapy lines were significantly associated with a significant better PFS. Response to therapy was the only prognostic predictive factor for OS. In the 60 patients analyzed for k-ras mutations, number of chemotherapy lines, increased EGFR-GCN and k-ras wild type status predicted a better PFS.

**Conclusion:**

In metastatic CRC patients treated with chemotherapy plus Cetuximab number of chemotherapy lines and increased EGFR-GCN were significantly associated with a better clinical outcome, independent of k-ras status.

## Introduction

Treatment of advanced colorectal cancer (CRC) patients in the last ten years rapidly moved from a single agent 5-fluorouracil (5-FU), modulated by Folinic Acid (FA), to combination chemotherapy including oxaliplatin (L-OHP) and irinotecan (CPT-11). The addiction of monoclonal antibodies directed to the vascular endothelial growth factor (VEGF), or to the epidermal growth factor receptor (EGFR) to a regimen with CPT-11-FA-5-FU increased progression free-survival (PFS) and overall survival (OS) in randomized phase III trials [1,2]. EGFR, whose locus is on the short arm of chromosome 7, is a transmembrane glycoprotein, with an intracellular tyrosine kinase domain. Binding of ligand to the EGFR domain induces receptor homodimerization or heterodimerization with other HER family members, which results in a transphophorilation of tyrosin-kinase and subsequent activation of a complex downstream signalling network [3]. EGFR activation appears to promote tumor growth and progression by controlling transcription, cell-cycle progression, apoptosis and differentiation [4]. Cetuximab is a MoAb active against the ligand binding site of EGFR with high specificity and higher affinity for EGF receptor than the natural ligands TGF-α and EGF. Preclinical models have demonstrated antitumor activity of Cetuximab by several mechanisms including inhibition of tumor cell proliferation, angiogenesis, invasion and potentiation of apoptosis; it seems also to mediate antibody-dependent cellular cytotoxicity [5]. In preclinical studies Cetuximab was able to overcome resistance to CPT-11 and to radiotherapy in colorectal cancer models [6,7]. Cetuximab is active either as a single agent and in combination with chemotherapy. Jonker et al. showed that Cetuximab increased PFS and OS when compared to best supportive care (BSC) in 572 patients previously treated with chemotherapy [8]. Cetuximab plus CPT-11 increased RR and PFS but not survival when compared to Cetuximab alone in the Bond trial [9] and to CPT-11 alone in the EPIC trial [10].

Phase II trials in untreated patients showed a high activity of the combination of Cetuximab plus doublets [11,12] or triplets [13]. A recent meta-analysis combining the OPUS and CRYSTAL trials showed an increase of overall survival adding Cetuximab to FOLFIRI (5-FU-FA-CPT-11) and FOLFOX_4_(5-FU-FA-L-OHP)[14]. When Pamitumumab, a fully humanized anti-EGFR anitibody, was added to FOLFOX_4 _in first line treatment or to FOLFIRI in second line treatment, it significantly increased RR and PFS [15,16]. The clinical relevance of these information indicate that chemotherapy plus an anti-EGFR antibody can be now considered as one standard option for patients with advanced CRC in first or second line of treatment. However these benefits are limited to a minority of patients and the identification of markers predictive of activity/resistance is clearly needed. EGFR expression, detected by immunohistochemistry (IHC), it does not represent a good predictive marker of response [17].

Moroni et al [18] were the first authors who evaluated the EGFR-gene copy number (GCN) in 31 selected patients with metastatic CRC treated with Cetuximab or Panitumumab. Eight out of nine patients who obtained a partial response had an increased EGFR gene copy number (GCN). By contrast, only one out of the twenty-one non-responders had an increased EGFR-GCN (p < 0.0001). However, there is no consensus on the predictive role of increased EGFR-GCN due to difficulty in reproducibility of the method of analysis, the limited number of patients evaluated and their heterogenic features. Lievre et al were the first who identified the mutation status of k-ras as the strongest predictive factor for resistance to anti-EGFR antibody showing that patients with mutated k-ras are genetically resistant to these agents. Therefore, the approved use of Cetuximab and Panitumumab is limited to patients with a wild-type k-ras status, because benefits in RR PFS and OS are limited only to k-ras wild-type patients.

The aim of the present study was to support further evidence of the predictive role of EGFR-GCN in terms of RR, PFS and OS in a retrospective series of 101 patients affected by advanced CRC and treated with chemotherapy plus Cetuximab. The role of kras status was also evaluated in a subset of 61 out of these 101 patients.

## Patients and Methods

### Patients eligibility

One hundred-one consecutive patients with pathologically confirmed metastatic CRC screened for EGFR immunostaining were retrospectively evaluated. Patients treated with Cetuximab as first line therapy had been previously included in controlled clinical trials, 2042 GOIM [12] and POCHER study [13] respectively. Pretreated patients received Cetuximab with CPT-11 alone or with FOLFIRI. Only one patients with pelvic recurrence and lung metastases was treated with Cetuximab and received a single course of radiotherapy.

Eligibility criteria included: age ≥ 18 years, Eastern Cooperative Oncology Group performance status of 0,1-2; life expectancy of at least 3 months; normal hematopoietic, hepatic, and renal functions; no history of brain metastases and no prior treatment with EGFR-targeting agents. Patients gave written informed consent before treatment.

### Dosage and Drug Administration

Cetuximab was delivered with the same dosage and schedule both as single agent or in combination: a 2-hour intravenous infusion at 400 mg/m^2 ^followed by weekly 1-hour infusion of 250 mg/m^2^.

In the GOIM study Cetuximab was administrated in combination with FOLFOX_4_; whereas in the POCHER trial Cetuximab was added to chrono-IFLO (5-FU at the dose of 550 mg/m2/d × 4 days, L-OHP at 15 mg/m2/d × 4 days, FA 150 mg/m2/d × 4 and CPT-11 at 130 mg/m2/d1) with courses every 2 weeks.

Toxicity was graded according to the National Cancer Institute Common Toxicity Criteria (version 2.0).

### Pretreatment and Follow-Up Studies

History, physical examinations, and a safety assessment were performed pre-treatment and weekly thereafter. Electrolytes, serum chemistries, liver and kidney function examinations were performed at baseline, every 2 weeks and at the end of treatment.

Tumors were measured pre-treatment and every 6 weeks and tumor response was assessed with CT scan according to the RECIST criteria [19].

#### EGFR Immunohistochemistry

Immunohistochemical stains were performed on 5 μ paraffin embedded tissue sections. Sections were deparaffinized and rehydrated in a series of alcohols and xylene according to established procedures. The sections were immunostained for EGFR using DAKO EGFR PharmDX kit (Dako, Milan, Italy). Antigen retrieval was performed using proteinase K for 5 min. Sections were then visualized with 3,3'-diaminobenzidine (DAB) as chromogenic substrate and counterstained with Mayer's haematoxylin. Negative controls included replacement of the primary antibody with non-reacting antibodies.

EGFR expression is defined positive as any membrane staining above background level was visualized. Moreover, an intensity score was applied as follows: negative no reaction; 1+ if the neoplastic cells displayed an incomplete, weak plasmamembrane/cytoplasmic; 2+ if neoplastic cells displayed a complete plasmamembrane immunostaining with a moderate intensity; and 3+ if neoplastic cells displayed a complete plasmamembrane strong immunostaining. Evaluation of the immunohistochemical results was performed independently and in blinded manner by two investigators (MM, MGD).

#### FISH analysis

The fluorescent-labeled probes used in the present study were LSI EGFR (Spectrum Orange), specific for the EGFR human gene locus (7p12) and the chromosome enumeration probe (CEP 7, Spectrum Green) for alpha-satellite DNA located at the centromere (7p11.1-q11.1) (Vysis, Downers Grove, IL). The assay was performed according to the manifacturer's instructions. In brief, the target DNA were heat-codenatured (2 minutes at 72°C) with probe mixtures and hybridized overnight at 37°C, using a Vysis Hybrite system. After hybridization for ~16 hours, hybridized samples were washed in 0.4× standard saline citrate (SSC)-0.3% NP40 at 73°C for 2 minutes and 2× SSC-0.1% NP40 at room temperature for 1 minute, Nuclei were counterstained with 4',6-diamidino-2-phenylindole (DAPI II).

Two hundred nuclei per specimen were observed using a fluorescence microscope with a 100× lens using an Olympus BX 61 fluorescence microscope equipped with a 100 watt mercury lamp and with the Triple Bandpass Filter set (Vysis) for DAPI, SpectrumOrange and SpectrumGreen. Fluorochrome signals were captured individually and images were generated via a computer with Quips genetic workstations and imaging software (Vysis).

EGFR gene was visualized as a red signal and the CEP 7 was visualized as a green signal. EGFR gene status was scored as the average number of EGFR red signals per nucleus and as the ratio between EGFR red signals and CEP7 green signals. Centromeric enumeration probe CEP7 was used as a control to determine copy number of chromosome 7, to adjust for the effects of aneuploid chromosome 7 when the *EGFR gene *copy numbers were counted Only nuclei with unambiguous chromosome 7 centromeric hybridization signals were scored for the EGFR signal numbers.

Polysomy of EGFR gene consisted of an increase of EGFR red signals (≥ three signals per nucleus) paralleled by the same increase of chromosome 7 (on which the EGFR gene is located) as measured by the number of CEP7 green signals per nucleus in at least 50% of neoplastic cells. Samples with a ratio EGFR gene/CEP7 ≥ 2.0 were esteemed amplified whereas samples displaying a CEP7 ≥ 3 were defined polysomic.

### DNA extraction and k-ras mutation analysis

DNA was extracted from 10 μ paraffin-embedded tumor sections after macrodissection using the DNA extraction kits QIAmp DNA kit (Qiagen-Explera, Jesi, Italy). according to the manufacturer's instructions. About 100-200 ng of genomic DNA was used in a PCR to amplify the region of exon 2 of K-Ras containing codon 12 an 13. The PCR reaction was as follows: 95°C for 5 min, 35 cycles of 94°C for 10 sec, 58°C for 10 sec, 72°C for 1 sec, and 72°C for 2 min. The PCR reaction buffer (KAPA2 Fast Hot start, Resnova, Genzano di Roma, Italy) out in a volume of 50 μl contained buffer A 1×, 200 μM dNTPs, 10 pmol Ki-ras sense primer (5'-AGGCCTGCTGAA AATGACTGAATA-3'), 10 pmol K-ras antisense primer (5'-CTGTATCAAAGAATGGTCCTGCAC-3'), and 1 U of Taq polymerase (Resnova). An additional no-template control containing only mix was run for every PCR reaction.

The PCR products were purified using Nucleospin Extract II Purification kit (M-Medical). Cycle sequencing was performed using BigDye Terminator v 3.1 kit (Applied Biosystems, Monza, Italy), and analyzed with a ABI 3130 capillary electrophoresis system (Applied Biosystems).

The presence of an heterozygous k-ras mutation in the tumor was defined as the appearance of a mutant peak with an height of at least one-third of that of the wild type. All sequencing analyses were performed at least twice on two independents PCRs.

In all the 61 CRC patients analysed, DNA was extracted from the primary tumour.

### Statistical Analysis

Descriptive statistics were used to summarize pertinent study information. The association between variables was tested by the Pearson Chi-Square test or the Fisher's Exact test. Logistic regression multivariate analysis was used to assess the impact of the following variables on the response rate: number of lines, EGFR IHC score, GCN, number of metastatic lines, liver metastases, primary tumor site. Results are reported as odd ratio (OR) with 95% CI. PFS and OS were calculated by the Kaplan-Meier product-limit method from the date of the first day of treatment until progression of disease or death for any cause or for disease. If a patient had not progressed/died, survival or progression was censored at the time of the last visit. The log-rank test was used to assess differences between subgroups. Significance was defined at the p < 0.05 level [20]. The Hazard risk and the confidence limits were estimated for each variable using the Cox univariate model and adopting the most suitable prognostic category as referent group [21]. A multivariate Cox proportional hazard model was also developed using stepwise regression (forward selection) with predictive variables which were significant in the univariate analyses. Enter limit and remove limit were p = 0.10 and p = 0.15 respectively. The SPSS (13.0) statistical program was used for analysis.

## Results

### EGFR Protein Expression and EGFR-GCN

EGFR protein was over-expressed in 90 out of 101 patients (89%), 22 with a 1+ staining score, 40 with a 2+ score and 28 with a 3+ score.

An increased EGFR-GCN was present in 60/101 (59%) patient tumor samples. Gene amplification was seen only in 4/101 patient tumor samples (4%) as previously reported [22] (Table 1). There was no correlation between intensity of EGFR IHC score and increased EGFR-GCN by FISH (p = 0.43).

**Table 1:** FISH Data

Patterns FISH	Patients	%
Increased EGFR gene copy number in >50% of cells	56/101	56
EGFR gene copy number <40% of cells	28/101	28
EGFR gene amplification	4	4
Not evaluable	13	12

### Response to Chemotherapy plus Cetuximab

From February 2004 to May 2007 101 CRC patients (62 male, 39 females; median age 63 years, range 26-80) were screened for EGFR tumor expression and treated with Cetuximab (Table 2). Forty-three patients were treated with chemotherapy plus Cetuximab as first line. Fifty-eight patients received Cetuximab as second or more lines of chemotherapy with a median number of two (range 1-5) and a median interval of 18 months (range 1-60) between starting of chemotherapy and Cetuximab. Only 12 patients received Cetuximab as monochemotherapy.

**Table 2:** Patient characteristics

**Patient characteristics**	**Number**	**%**
		
Total evaluated	101	100
		
Gender		
Male	62	61
Female	39	39
		
Median age (years range)	63(26-80)	
		
Performance Status		
0	79	78
1	20	20
>1	2	2
		
Primary tumor site		
Colon	79	78
Rectum	22	22
		
Liver metastases	79	78
		
Number of metastatic sites		
1	77	76
>1	24	24
		
Previous chemotherapy lines		
0	43	42
1-2	40	40
>2	18	18
		
Median number of previous lines (range)	2 (1-5)	
		
Median interval time between first-line treatment and Cetuximab (months, range)	18 (1-60)	
		
Type of chemotherapy associatad with Cetuximab		
CPT-11	19	19
CPT-11, L-OHP, 5-FU	29	29
FOLFIRI	21	21
FOLFOX	20	20
Only Cetuximab	12	12

Ninety-nine out of 101 patients were evaluable for response: 40 patients (40%, CI 31-50) had a partial response, 31 (31%) had a stable disease, 20 patients (20%) had a progression and eight (8%) had non-measurable disease. In those patients who received Cetuximab as first line treatment we observed a RR of 70% (30/43) while it reached 18% (10/56) in the group with 2^nd ^or further line of therapy (p < 0.0001).

Response was observed in 29/60 (48%) of patients with increased EGFR-GCN and in 6/28 (21%) in those without increased EGFR-GCN (p = 0.02); 13 patients were not evaluable at FISH analysis.

Multivariate regression analysis showed that patients treated as first line had a better chance of response than pretreated patients [HR 13.90 (4.41-43.83) p < 0.0001] and those with increased EGFR-GCN better than non-increased [HR 6.27 (1.72-22.89) p < 0.005].

### Relation between EGFR-GCN and Protein Expression with PFS and OS

At time analysis was done 65 patients (64%) progressed and only 19 patients (19%) deceased. Median follow-up for all patients was 12 months (range 1-34).

In the group of patients as first line treatment median PFS was 12 months (95% CI 9-15) versus a median PFS of 6 months (95% CI 4-9) for the group of patients who received Cetuximab as a II or more line therapy, p = 0.01.

**Figure 1:**
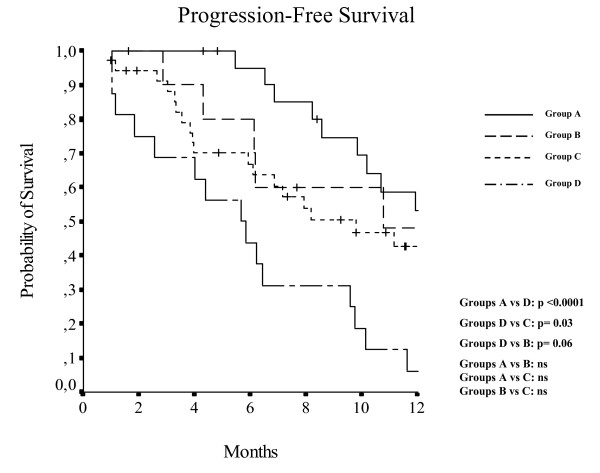
**PFS in four group of patients**. *For corresponding lines see the key in figure 1*. Group A: +GCN/1st line; Group B: -GCN/1st line; Group C: +GCN/more lines; Group D: - GCN/more lines; (+: increased; -: non-increased; GCN: EGFR Gene Copy Number) P value adj: Groups A vs D: p < 0.0001; Groups D vs C: p = 0.03; Groups D vs B: p = 0.06; Groups A vs B: ns; Groups A vs C: ns; Groups B vs C: ns.

As illustrated in Table 3, Cox model analysis showed IHC EGFR score 2-3 increased EGFR-GCN and first line chemotherapy significantly associated with a better PFS. When patients were divided into four groups, according to line of therapy and EGFR-GCN, a statistically significant difference for PFS was observed, with first-line patients/increased EGFR-GCN having the best PFS and pre-treated/non-increased EGFR-GCN the worst (p < 0.0001) (Figure 1).

**Table 3:** Multivariate analysis for Progression Free Survival

Variables	Entire Population 101 patients		K-ras Population 61 patients	
	HR (CI 95%)	p value	HR (CI 95%)	p value
Increasd EGFR-GCN: no vs yes	1.84 (1.07 - 3.15)	0.03	2.20 (1.03 -- 4.70)	0.04
Number of Lines: II-III vs I	2.20 (1.25 - 3.89)	0.01	4.60 (1.87 -- 11.31)	0.001
EGFR score: 0-1 vs 2-3	2.27 (1.35 - 3.82)	0.002	1.99 (0.89 -- 4.44)	0.09
k-ras mut vs wt	-	-	2.14 (0.97 -- 4.73)	0.06

Mut: mutant; wt: wild-type

At multivariate analysis response to therapy was the only prognostic predictive factor for OS. No difference in OS was observed among the four groups of patients (data not shown).

### K-ras analysis and EGFR-GCN

k-ras analysis was performed in 61/101(59.4%) patients. There were no differences in clinical data, in patients with or without K-ras analysis, such as median age, EGFR expression, EGFR-GCN, site of primary tumor, incidence of liver metastasis, response to treatment and line of chemotherapy treatment (data not shown). There was no correlation between patients with increased EGFR-GCN and wild-type status 23/35 (65%) and patients with non-increased EGFR-GCN and wild-type status 11/18 (61%).

K-ras mutations were found in 23/61 (37.7%). There was no correlation between k-ras status and response to treatment with 18/38 objective response (47.4%) in k-ras wt patients and 10/22 (45,5%) in k-ras mutation, with an OR = 1.08 [(CI 95% 0.38-3.10), p = 0.89]. One patient was not evaluable for response. In the 61 patients analyzed for k-ras increased EGFR-GCN maintained its predictive role for PFS together with EGFR-score 2-3 and k-ras status wild-type (Table 3).

## Discussion

The availability of anti-EGFR antibodies in advanced colorectal cancer and the need to increase their limited efficiency in unselected patients are factors requiring the development and validation of laboratory tests which could predict who might benefit from this treatment in terms of activity and efficacy.

We studied a population of patients mainly treated with chemotherapy plus Cetuximab, 42% of whom treated as first line treatment. We demonstrated that: a) there is no correlation between EGFR immunostaining and EGFR-GCN; b) increased EGFR-GCN, first line chemotherapy, EGFR score 2-3 were predictive factors for PFS; c) these data were confirmed as independent from k-ras status.

We are aware that interpretation of these results seem to be difficult because of a mixed sample of patients treated as first or further lines of chemotherapy and mostly having received chemotherapy plus Cetuximab and not Cetuximab alone.

However the increase of RR due to Cetuximab addiction in CPT-11 containing regimens in first or second line of therapy is in the same range. In the Crystal trial RR was 59.3% with FOLFIRI plus Cetuximab versus 43.2% in FOLFIRI alone (wild type patients) [2]. In the EPIC trial RR was 4.2% with CPT-11 and 16.4% with CPT-11 plus Cetuximab [10]. In The Peeters trial [16] in second line of treatment RR was 10% with FOLFIRI alone and 35% FOLFIRI plus Panitumumab. So it could be reasonable to analyse patients in first or more line of chemotherapy together.

The role of increased EGFR-GCN and of number of chemotherapy lines as prognostic factors in the Kaplan-Meier curves for PFS are clearly shown in Figure 1 where four distinct groups of patients can be separated according to the line of chemotherapy and EGFR-GCN. Patients treated as first line and with an increased EGFR-GCN had the best PFS but a significant difference in PFS was also found in patients treated with Cetuximab plus chemotherapy as second or more line with or without an increased EGFR-GCN (p = 0.03).

In our population 89/101 patients were treated with combination of chemotherapy plus Cetuximab. Literature concerning the role of EGFR-GCN in patients treated with chemotherapy alone is very limited. Our data indicate that, in the context of combination of chemotherapy plus an anti-EGFR antibody, the assessment of EGFR-GCN can be a valuable tool for better selecting potential responding patients. The recent survival gain in the Crystal Study [14] will increase the number of k-ras wild type patients treated simultaneously with both therapeutic agents. Our results are further supported by the evidence that an increase of EGFR-GCN had a significant positive impact on PFS independently of k-ras status.

Recently Moroni et al. have highlighted the most relevant elements of the clinical significance of EGFR FISH in CRC [23]. According to this author reproducibility remains a large obstacle for its practical usefulness. However when we look at the different cut-off values in the literature they do appear not to differ significantly. Using FISH Sartore-Bianchi identified GCN ≥ 2,5/nucleus or Chromosome 7 polysomy or amplification ≥ 40% of neoplastic cells [24], Cappuzzo et al used GCN>2.92 and found a significant relationship with RR and PFS but not with OS [25], Personeni et al defined GCN as ≥ 2.83 and confirmed a relationship with RR and OS [26]. In our series a sample was defined polysomic for the EGFR gene when at least 50% of examined neoplastic cells had ≥ 3 signals per nucleus paralleled by the same increase of chromosome 7 on which the EGFR gene is located. Therefore, much of the data from literature is similar although an international consensus on the definition of cut-off points is needed.

Is the information coming from GCN useful in clinical practice for the patient? EGFR-GCN is indicative of a subgroup of patients who will most likely benefit from this combination but currently FISH results do not enable us to discriminate the responsive patient.

The molecular picture of colorectal cancer seems to be so complex that it is difficult to identify a single molecular marker to assess responsiveness or a better outcome. K-ras mutation has emerged as the strongest predictive factor for resistance to anti-EGFR moAbs [27] but its role as a marker of survival has been demonstrated only in wild-type and not in the mutated patients when treated with panitumumab [28]. Recent data from Crystal and OPUS studies showed that addiction of Cetuximab to FOLFIRI (183 patients) versus FOLFIRI alone (214 patient) was associated with a non statistically significant increase in RR, PFS, and OS in k-ras mutant patients [14]. Etienne-Grimaldi et al showed that advanced CRC patients treated with 5-FU without anti-EGFR moAb had the same response potentiality and the same survival rates independent from k-ras mutational status [29].

Another point to be addressed is the role of IHC for EGFR. To date, it has been demonstrated that the tumor EGFR expression detected by IHC does not represent a good predictive marker of response to Cetuximab. The analysis of our results showed that the intensity of EGFR tumor expression (IHC score 2-3 vs 0-1) was significantly related to a prolonged PFS (Table 3). At our knowledge, this is the first study in which EGFR overexpression (score 2+/3+) detected by IHC appears to be relevant in predicting PFS demonstrating that patients bearing advanced CRC strongly positive for EGFR may benefit from therapy with MoAbs. Up to now, we have no explanation for this result which is contrary to that reported in the literature and needs to be confirmed in a larger and more homogeneous series.

In conclusion, in our advanced CRC population treated with Cetuximab plus chemotherapy an increased EGFR-GCN conferred a treatment advantage in untreated and pretreated patients. This effect was maintained in the subset of k-ras evaluated patients. Integration of this information with that coming from other molecular pathways could lead to a personalized "targeted" therapy for these patients.

## Competing interests

The authors declare that they have no competing interests.

## Authors' contributions

CC: study design, acquisition, analysis and interpretation of data; drafting the manuscript. MM: the EGFR IHC analysis; drafting the manuscript. AC: FISH analysis. AT: involved in acquisition and interpretation of data; drafting the manuscript; RM: FISH analysis. IS: statistical analysis. EM: DNA extraction and k-ras mutation analysis. SC: DNA extraction and k-ras mutation analysis. MGD: pathological sample evaluation. MZ: acquisition of data. GP: in acquisition of data. FC:drafting the manuscript; CG: study design, acquisition, analysis and interpretation of data; drafting the manuscript. All authors read and approved the final manuscript.
